# Wait Times Experienced by Lung Cancer Patients in the BC Southern Interior to Obtain Oncologic Care: Exploration of the Intervals from First Abnormal Imaging to Oncologic Treatment

**DOI:** 10.7759/cureus.330

**Published:** 2015-09-22

**Authors:** David Van de Vosse, Rezwan Chowdhury, Andrew Boyce, Ross Halperin

**Affiliations:** 1 Department of Radiation Oncology, BC Cancer Agency, Sindi Ahluwalia Hawkins Centre for the Southern Interior

**Keywords:** lung cancer, time interval, wait time, outcomes

## Abstract

Background: Lung cancer is associated with rapid disease progression, which can significantly progress over a duration of four to eight weeks. This study examines the time interval lung cancer patients from the interior of British Columbia (BC) experience while undergoing diagnostic evaluation, biopsy, staging, and preparation for treatment.

Methods: A chart review of lung cancer patients (n=231) referred to the BC Cancer Agency Centre for the Southern Interior between January 1, 2010 and December 31, 2011 was performed. Time zero was defined as the date of the first abnormal chest imaging. Time intervals, expressed as median averages, to specialist consult, biopsy, oncologic referral, initial oncology consultation, and commencement of oncologic treatment were obtained.

Results: The median time interval from first abnormal chest imaging to a specialist consultation was 18 days (interquartile range, IQR, 7-36). An additional nine days elapsed prior to biopsy in the form of bronchoscopy, CT-guided biopsy, or sputum cytology (median; IQR, 3-21); if lobectomy was required, 18 days elapsed (median; IQR, 9-28). Eight days were required for pathologic diagnosis and subsequent referral to the cancer centre (median; IQR, 3-16.5). Once referral was received, 10 days elapsed prior to consultation with either a medical or radiation oncologist (median, IQR 5-18). Finally, eight days was required for initiation of radiation and/or chemotherapy (median; IQR, 1-15). The median wait time from detection of lung cancer on imaging to oncologic treatment in the form of radiation and/or chemotherapy was 65.5 days (IQR, 41.5-104.3).

Interpretation: Patients in the BC Southern Interior experience considerable delays in accessing lung cancer care. During this time, the disease has the potential to significantly progress and it is possible that a subset of patients may lose their opportunity for curative intent treatment.

## Introduction

Lung cancer represents a significant cause of morbidity and mortality in the province of British Columbia. Despite having the lowest mortality rates of lung cancer within Canada (BC, 38.1 per 100,000), the overall age-standardized five-year survival for lung cancer in BC is below the national average (BC, 16.9%, Canada, 18.4%) [1]. Albeit, this is similar to the United States (17.8%) and greater than the European average (13%) [2-3]. Accordingly, of the nearly 3,000 new lung cancer diagnoses in BC each year, approximately 2,250 deaths occur [4]. Overall, deaths attributable to lung cancer are greater than that of the next three leading causes (colorectal, breast, and prostate carcinomas) of cancer-related deaths combined [4]. The high mortality rate has been attributed to, among others, a late stage of disease presentation at the time of diagnosis and the rapid progression of lung cancer [5-6]. For instance, small-cell lung cancer exhibits a tumor-doubling time as rapid as 30 days (d) [6-7], suggesting that any delay between onset of symptoms, diagnosis, and oncologic treatment has the potential for significant disease progression and may preclude a subset of patients to lose their opportunity for curative intent [8-9]. If significant delays in care exist, a key strategy for improving outcomes would be to reduce the time interval lung cancer patients experience. Reducing wait times may be easier, and possibly more economically viable, than developing new drugs and treatments to delay or reverse disease course once it has advanced.  

Determination of timelines of care for lung cancer patients globally has revealed considerable variability [10-24]. For instance, in the United States, time intervals from initial abnormal radiograph to treatment initiation vary from 35 to 84 d [14, 16-17, 20]. While relatively few studies have been performed regarding lung cancer wait times within Canada [21-24], one prospective study in Ontario reported a median time interval from development of first symptoms to initiation of treatment of 138 d [23], while another study from Manitoba reported a median of 145 d from first physician visit to diagnosis [22]. Such differences in time intervals among various centres highlight the need for individual centres to perform internal quality assurance studies to identify avoidable delays in treatment.

The vast majority of studies examining lung cancer wait times have been conducted at centres servicing dense, urban municipalities. As a result, data on time intervals experienced by rural patients are lacking. The BC Cancer Agency Centre for the Southern Interior (BCCA-CSI) is distinct in that it services both urban and remote rural populations. Its catchment population of ~750,000 is distributed over an area approximately the size of the United Kingdom [25-26], stretching from the Alberta border to the east, the Fraser Valley to the west, Quesnel to the north, and the United States border to the south. Consequently, nearly one out of five patients must travel more than 300 km to access oncologic care, with some patients travelling up to 700 km. Considering the recent 2013 Organization for Economic Cooperation and Development (OECD) health report determined Canada has the third highest rate of lung cancer mortality among women in the developed world (47 per 100,000), almost double the OECD average (26.5 per 100,000) [27], a question arises whether this number is partly due to the performance of our health care system, and whether geographic distribution plays a significant role. In light of these considerations, we evaluated the time interval experienced by lung cancer patients referred to the BCCA-CSI. Our study provides data relating to wait times experienced by lung cancer patients outside of the traditionally studied metropolitan centres and provides evidence for a focused and detailed redesign of the cancer care pathway with emphasis on reducing delays at all stages of lung cancer management.

## Materials and methods

### Study design

A retrospective chart review was conducted of newly diagnosed lung cancer cases referred to a regional cancer centre (BCCA-CSI) serving both urban and rural communities within the Southern Interior of British Columbia. Data from 713 patients, referred over a two-year period (January 1, 2010 to December 31, 2011), were obtained from the BC Cancer Agency, Data Requests-Surveillance and Outcomes and supplemented by chart review of available electronic medical records accessed via Cancer Agency Information System (CAIS) software (BCCA-EMR). For each case, information was collected on date and type of diagnostic imaging; date and type of specialist consult; date and type of biopsy; histology and staging of disease; date of lobectomy, if applicable; date of referral to BCCA-CSI; date and type of oncologic consult; date and type of oncologic treatment; intent of treatment; date of death; and finally, the Health Service Delivery Area (HSDA) accessed.

This study was submitted to and approved by the British Columbia Cancer Agency’s research ethics review board (approval #H13-02129). Informed patient consent was obtained at the time of treatment.

### Primary outcomes and study population

The primary goal of this study was to determine time intervals between each of the following sequential chart entries: first abnormal diagnostic image, specialist consult (thoracic surgery, respirology, or other specialty), biopsy, referral to BCCA-CSI, oncology consultation (radiation or medical), and initiation of treatment (chemotherapy and/or radiation therapy). Time intervals were further divided on the basis of treatment intent, either curative or palliative, as charted by an oncologist, or distance from primary residence to BCCA-CSI. For cases to be included in this study, charts were required to contain dates for the following entries: initial abnormal diagnostic imaging, biopsy, specialist consult, referral to BCCA-CSI, oncologic consult, and treatment initiation. The typical lung cancer care path was defined as an abnormal diagnostic imaging result, followed by specialist consultation, biopsy, referral to BCCA-CSI, and oncologist consultation, and finally, treatment commencement. Certain groups of patients were excluded in five steps: 1) those with recurrent lung cancer, 2) those who did not follow the typical lung cancer care path, including requiring a second biopsy, 3) those who received care outside of the Southern Interior, or who voluntarily cancelled/delayed a scheduled appointment, 4) those with comorbidities or concurrent cancers deemed to significantly alter the typical treatment care path due to additional consultations and/or tests, and lastly, 5) those lacking information on one or more of the required dates. See Table 1 for additional exclusion criteria.

**Table 1:** Patient exclusion criteria

Exclusion Criteria	Number of Patients
Recurrent lung cancer	49
Non-typical cancer care path	15
Biopsy after referral	50
> 2 biopsies	19
Health care accessed outside of the health authority	31
Patient delay following abnormal diagnostic image	25
Patient delay following diagnosis	38
Significant comorbidity	17
Concurrent cancer	19
Death	1
Missing Information Regarding:	
Diagnostic imaging	22
Biopsy	23
Referral to cancer centre	15

Cancer care of the remaining patients were divided into two clinical care pathways: 1) a standard care pathway, representing 231 patients that followed the typical lung cancer pathway described above, and 2) an alternative care pathway, representing 158 patients who, following abnormal diagnostic imaging, circumvented specialist consultation, and proceeded directly to biopsy. The two cancer care pathways were analyzed in multiple time intervals (Tis) to break down the wait times for the various steps to treatment (Table 2). Ti-Two, the time intervals from specialist consult to biopsy, were subdivided according to the area of specialty: thoracic surgery, respirology within or outside Kelowna, or other specialties (i.e. neurosurgery, internal medicine). For Ti-Five and Ti-Six, only those patients who received treatment were included; consequently, sample sizes were reduced slightly for the standard care pathway (n=200) and alternative pathway (n=140). Ti-Six was further subdivided according to treatment intent, either curative or palliative. For each Ti, we calculated the median, mean, interquartile range, 90^th^ percentile, and range of values, expressed in days (d). Similar statistical analyses were performed to assess wait times according to the distance from primary residence to the BCCA-CSI.

**Table 2:** Time intervals examined

Time Intervals Examined	
Ti-One	Abnormal diagnostic imaging to specialist consult
Ti-Two	Specialist consult to biopsy
Ti-Three-A	Biopsy to cancer centre referral
Ti-Three-B	Biopsy to lobectomy, if required
Ti-Four	Cancer centre referral to oncologic consult
Ti-Five	Oncologic consult to first treatment
Ti-Six	Total time interval from abnormal image to first treatment

## Results

### Clinicopathologic characteristics

During the two-year study period, 713 newly diagnosed lung cancer patients were referred to the BCCA-CSI. Of these, 231 and 158 cases met our criteria for further analysis of the time interval experienced by lung cancer patients following the standard and alternative care pathways, respectively. Patient demographics for both care pathways are listed in Table 3. Age and gender data were not collected.

**Table 3:** Patient demographics

	Standard Care Pathway			Alternative Care Pathway	
Variable	n = 231	%		n = 140	%
*Health Service Delivery Area*					
Okanagan	137	59.3		66	47.1
Thompson-Cariboo	42	18.2		33	23.6
Kootenay Boundary	31	13.4		14	10.0
Northern Interior	6	2.6		5	3.6
East Kootenay	5	2.2		17	12.1
Northeast	1	0.4		1	0.7
Fraser East	1	0.4		1	0.7
Unspecified	8	3.5		3	2.1
*Specialist Consultation*					
Thoracic	99	42.9		-	-
Respirology in Kelowna	68	29.4		-	-
Respirology outside Kelowna	38	16.5		-	-
Other (Int. Med., Neuro Surg.)	26	11.3		-	-
*Biopsy*					
Bronchoscopy	141	61.0		62	44.3
CT-guided	47	20.3		45	32.1
Fine needle aspiration	24	10.4		24	17.1
Thoracentesis	4	1.7		2	1.4
Mediastinoscopy	4	1.7		-	-
Sputum	3	1.3		3	2.1
Craniotomy	3	1.3		-	-
PET	2	0.9		-	-
Laminectomy	1	0.4		-	-
Lobectomy	1	0.4		-	-
Metastasis	1	0.4		4	2.9
*Histology*					
NSCLC	177	76.6		107	76.4
SCLC	48	20.8		20	14.3
Mesothelioma	5	2.2		3	2.1
Unspecified	1	0.4		10	7.1
*Staging*					
IA	8	3.5		9	6.4
IB	11	4.8		2	1.4
IIA	11	4.8		6	4.3
IIB	7	3.0		6	4.3
IIIA	33	14.3		25	17.9
IIIB	22	9.5		14	10.0
IV	68	29.4		55	39.3
Limited stage SCLC	10	4.3		7	5.0
Extensive stage SCLC	28	12.1		4	2.9
Unknown	33	14.3		12	8.6
*Treatment Intent*					
Curative	58	25.1		36	25.7
Palliative	173	74.9		104	74.3

Approximately half of patients, regardless of the care pathway, accessed care in the Okanagan HSDA, encompassing the urban municipalities of the Okanagan Valley: Kelowna, Penticton, and Vernon. Approximately one-quarter of patients accessed care in the Thompson-Cariboo HSDA, comprising the city of Kamloops and surrounding rural communities, containing both urban and rural patients. The remaining patients accessed care in HSDAs consisting predominantly of small, rural communities.

Following abnormal diagnostic evaluation, referral to a thoracic specialist was the most frequent consultation (42.9%) and referral to a respirologist within Kelowna the second most frequent (29.4%). A small cohort (11.3%) of patients received a specialist consultation outside of respirology or thoracic surgery, predominantly in internal medicine or neurosurgery.

Bronchoscopy was the most common form of biopsy (61% of standard care pathway: 44% alternative care pathway), followed by CT-guided biopsy (20.3% and 32.1%), and fine needle aspiration (10.4% and 17.1%), in the standard and alternative care pathways, respectively. Histologically, the two pathways were similar with the vast majority of patients diagnosed with non-small cell lung carcinoma (NSCLC, 76%) most of whom, in agreement with previous studies, had advanced disease (Stage III or greater) [5]. However, the alternative care pathway had a higher incidence of unspecified histology (7.1% compared to 0.4%) and a lower rate of small cell lung carcinoma (SCLC) at 14.3% versus 20.8%.

Interestingly, individuals within the alternative pathway who bypassed a specialist consultation following abnormal diagnostic imaging were more likely to be diagnosed with either early stage (Stage 1A; 6.4% alternative pathway versus 3.5% standard pathway) or late stage of disease (Stage IV; 39.3% versus 29.4%) than the standard care pathway. These results are consistent with a practice in which patients presenting with symptoms of advanced disease are expedited in order to obtain palliative treatment. Similarly, patients presenting with localized, early stages of disease with a significant chance of curative intent may also be expedited. Despite this, however, the percentage of patients receiving treatment with curative intent was almost identical between the two pathways (25.1% standard; 25.7% non-standard).

### Time intervals

For each time interval, the median, mean, interquartile range, 90^th^ percentile, and range of values are presented in Tables 4 and 5. The median wait time from first abnormal chest imaging to specialist consultation, i.e., Ti-One was 18 days (d). Ti-One was further analyzed according to the type of specialist consultation. Wait times were longest for patients who underwent a thoracic surgery consultation, with a median wait of 22 d. Median wait time for patients who underwent a respirology consultation was similar at 20.5 d if the appointment occurred outside Kelowna, versus 10.5 d if the appointment occurred in Kelowna. Patients who obtained a consultation outside of thoracic surgery or respirology (i.e. neurosurgery, internal medicine) experienced the shortest time interval, a median wait of 7.5 d.

**Table 4:** Time intervals for standard care pathway

Interval		Median	Interquartile Range	90^th ^Percentile	Mean	Range	n
Ti-One	Abnormal image to specialist consult	18	7 - 36	61	24.3	0 - 181	231
	Thoracic surgery	22	10 - 36	62.2	26.9	0 - 85	99
	Respirology urban (Kelowna)	10.5	5 - 36.3	53.8	21.6	0 - 83	68
	Respirology rural (outside Kelowna)	20.5	7 - 41.8	61	29.0	0 - 181	38
	Other (Internal Med., Neurosurgery)	7.5	2.3 - 20	35.5	14.8	0 - 76	26
Ti-Two	Specialist consult to biopsy	9	3 - 21	30	13.9	0 - 113	231
	Thoracic surgery to bronchoscopy	20.5	9 - 28	35.6	21.8	0 - 113	68
	Respirology (urban) to bronchoscopy	4.5	1 - 7.3	20.5	8.1	0 - 54	44
	Respirology (rural) to bronchoscopy	6	2 - 9	22.4	8.1	0 - 30	20
	Other specialist to bronchoscopy	5	0 - 15	19.4	7.6	0 - 21	9
Ti-Three-A	Biopsy to cancer centre referral	8	3 - 16.5	40	14.7	0 -126	231
Ti-Three-B	Biopsy to lobectomy	18	9 - 28	53.2	24.1	0 - 92	17
Ti-Four	Cancer centre referral to oncologic consult	10	5 - 18	24	11.9	0 - 47	231
Ti-Five	Oncologic consult to first treatment	8	1 - 15	30	12.5	0 - 118	200
Ti-Six	Abnormal image to first treatment	65.5	41.5 - 104.3	136.2	74.5	2 - 254	200
	Palliative intent	57.5	35.5 - 92.5	131	67	2 - 254	148
	Curative intent	91	62 - 133	165.2	96	10 - 205	52

**Table 5:** Time intervals for alternative clinical pathway

Time Interval		Median	Interquartile Range	90^th ^Percentile	Mean	Range	n
Ti-Four	Cancer centre referral to oncologic consult	9	4 - 15	21	10.6	0 - 35	140
Ti-Five	Oncologic consult to first treatment	7	1 - 15.3	24.1	10.3	0 - 76	140
Ti-Six	Abnormal image to first treatment	55.5	22 - 89.5	134.4	64.1	1 - 320	140
	Palliative intent	46	20 - 66.3	102.7	49.8	1 - 153	104
	Curative intent	93.5	63.5 - 139	169	101.7	7 - 315	36

A median wait of 9 d elapsed between specialist consultation and biopsy (Ti-Two) in the form of bronchoscopy, CT-guided biopsy, or sputum cytology; if lobectomy was required, a median of 18 d elapsed from the time of biopsy to lobectomy (Ti-Three-B; Table 4). Subdividing the time interval from specialist consultation to bronchoscopy according to specialist type revealed a median wait-time of 20.5 d from thoracic surgery. By contrast, only 4.5 d elapsed from respirology consultation within Kelowna and 6 d if respirology consultation occurred outside Kelowna. A similar time interval (5 d) to bronchoscopy was observed following consultation with neurosurgery or internal medicine. A median of 8 d was required for pathological diagnosis and subsequent referral to the BCCA-CSI (Ti-Three-A). Once referral was received, an additional 10 d (median) passed before consultation with either a medical or radiation oncologist (Ti-Four) and finally, a further 8 d elapsed before patients received their first treatment in the form of radiation and/or chemotherapy (Ti-Five). For those patients following the alternative care pathway, similar wait times were observed for time intervals Ti-Four and Ti-Five (Table 5).

Overall, the median wait time from detection of lung cancer on imaging to radiation and/or chemotherapy treatment for patients following the standard care pathway was 65.5 d (mean 74.5, n=200), or approximately 9.5 weeks (Ti-Six; Table 4). Moreover, 25% of patients waited greater than 104.3 d. Subdividing Ti-Six by treatment intent revealed a median elapsed time of 57.5 d for patients obtaining palliative treatment versus 91 d for patients receiving treatment with curative intent. Of those patients receiving curative treatment, 25% waited longer than 133 d (4.5 months) and 10% waited longer than 165 d (5.5 months).

The median time interval of 55.5 d from abnormal diagnostic imaging to first treatment (Ti-Six; Table 5) for the alternative care pathway was 10 days shorter than the standard care pathway, presumably due to the absence of a specialist consultation. This interval was reduced to 46 d for patients requiring palliative treatment but increased to 93.5 d for those requiring curative treatment.

Due to the poor prognosis associated with a lung cancer diagnosis, sufficient time had elapsed to permit assessment of overall survival within the study population. At the time of analysis, 87% and 89% of patients were deceased in the standard and alternative pathways, respectively (data not shown). Survival was calculated from the date of abnormal diagnostic imaging to the date of analysis, resulting in a median survival of 315.5 d for patients proceeding along the standard care pathway (Table 6). Patients who received palliative treatment had a considerably lower median survival (234.5 d) than those treated with curative intent (976 d). Additionally, patients treated in the alternative care pathway had a lower median survival than patients treated in the standard care pathway (269 d versus 315.5 d).

**Table 6:** Survival in days from abnormal diagnostic imaging

Pathway	Median	Interquartile Range	90^th ^Percentile	Mean	Range	n
Standard care pathway	315.5	132 - 698	1121.3	473	30 - 1776	200
Palliative intent	234.5	106 - 416	744.6	322	30 - 1771	148
Curative intent	976	521 - 1226	1529.6	904	73 - 1776	52
Alternative care pathway	269	124 - 610	1128.1	645	21 - 1617	140
Palliative intent	209	94 - 420	800.7	315.6	21 - 1361	104
Curative intent	712	348 - 1202	1410	759	56 - 1617	36

### Distance from cancer centre

Analysis of wait times amongst the different HSDAs indicated that, in general, patients in the standard care pathway residing further from the cancer centre received treatment faster. For instance, patients residing within 20 km of the BCCA-CSI waited a median of 69 d (Ti-Six) for treatment while patients residing >300 km began treatment 9 d sooner (60 d; Table 7). Notably, this was not the case for patients in the alternative pathway, in which those residing at extreme distances, <20 km or >300 km, experienced a shorter time interval (~38 d) than those residing at intermediate distances (60-65 d). Together, these results suggest that, despite the remoteness of many communities in relation to the BCCA-CSI, rural patients, in fact, often receive care more promptly than their urban counterparts.

**Table 7:** Time elapsed in days from abnormal image to first treatment according to distance from BCAA CCSI (Interior Health Patients only)

Distance from BCAA CSI	Median	Interquartile Range	90^th ^Percentile	Mean	Range	n
Standard care pathway						
< 20 km	69	38.3 - 107.5	140.5	73.9	6 - 205	62
20 - 99 km	66	43.5 - 105	132	74.9	2 - 167	51
100-299 km	57	38.5 - 99	132.1	69.6	3 - 193	60
> 300 km	60	44 - 100.5	133.5	72.9	10 - 192	22
Alternative care pathway						
< 20 km	38	17.5 -58	101.7	52.8	12 - 207	24
20 - 99 km	60	22.3 - 90.5	125.1	63.2	7 - 156	34
100-299 km	65	40 - 97	124	70.2	6 - 216	41
> 300 km	38.5	15.3 - 86.8	144.2	60.8	1 - 320	34

### Time intervals according to Health Service Delivery Area

Generally, patients from the predominantly urban Okanagan HSDA experienced an overall time interval (Ti-Six) similar to the median corresponding to the care pathway undertaken (Table 8). In contrast, the small cohort of patients residing in the Northern Interior who sought treatment at the BCCA-CSI experienced the longest time interval (106 d; n=6), nearly twice that of those residing in the Thompson-Cariboo (57 d; n=39). Surprisingly, those patients from the more remote and rural HSDAs (East Kootenay and Kootenay Boundary) exhibited contrasting results. Under the standard care pathway, a time of 95 d elapsed for the Kootenay Boundary patients whereas only 23 d elapsed for the East Kootenay patients. Interestingly, these time intervals were reversed for patients accessing the alternative care pathway, 21.5 d and 55 d for patients of Kootenay Boundary and East Kootenay HSDA, respectively. These results likely reflect the relatively small sample size for these two regions; however, they may also represent differences in the management of lung cancer patients within the two HSDAs.

**Table 8:** Time elapsed in days from abnormal image to first treatment according to Health Delivery Services Area (HSDA)

HSDA	Median	Interquartile Range	90^th ^Percentile	Mean	Range	n
Standard care pathway						
Okanagan	68	40 - 102	138	74.2	2 - 205	117
Thompson-Cariboo	57	48.5 - 91.5	117	71.4	15 - 193	39
Kootenay Boundary	95	50.5 - 133	144.7	89.5	3 - 192	24
Northern Interior	106	98 - 110.3	182.5	117.2	30 - 254	6
East Kootenay	23	21 - 53	60.2	35	13 - 65	5
Northeast	99	N/A	N/A	99	N/A	1
Fraser East	138	N/A	N/A	138	N/A	1
Unspecified	15	7.5 - 31.5	60.2	26	6 - 83	7
Alternative care pathway						
Okanagan	56	22.8 - 87.5	122.6	61.6	7 - 207	68
Thompson-Cariboo	65	40 - 98	125.6	71.6	10 - 216	33
Kootenay Boundary	21.5	20 - 38.5	70.8	31.6	6 - 88	14
Northern Interior	117	48 - 134	150.8	99.2	35 - 162	5
East Kootenay	55	12 - 130.3	146.6	76	1 - 320	18
Northwest	48	N/A	N/A	48	N/A	1
Fraser North	68	N/A	N/A	68	N/A	1

## Discussion

### Summary of results

Herein, we report on 200 lung cancer patients in the BC Southern Interior, diagnosed between 2010 and 2012, who experienced wait times of considerable length (median 65.5 days) between first abnormal imaging and initiation of oncologic treatment. Moreover, 25% of patients waited longer than 104.3 days (d). Further analysis showed that patients treated with curative intent waited a median of 91 d. Strikingly, 25% of curative intent patients waited greater than 133 d (~4.5 months) and 10% waited greater than 165 d (~5.5 months). In addition, we identified a further 140 patients who proceeded on an alternative care pathway, resulting in a slightly reduced wait time (median 55.5 d), presumably due to the absence of a non-oncologist, specialist consultation following an abnormal diagnostic image.

As noted earlier, the BCCA-CSI services a geographically dispersed population. Consequently, many of the referrals must travel long distances (up to 700 km) for diagnostic evaluation, specialist consultation, and oncologic treatment. Importantly, wait times for patients residing farther than 300 km from the cancer centre were equal to (alternative pathway) or shorter (standard pathway) than those experienced by patients residing within 20 km of the cancer centre, suggesting that travel distance is not a significant factor in timely access to cancer care in the BC Southern Interior.

### Previous literature

Several recent studies have examined the possibility that delays in care are contributing factors to the poor prognosis of lung cancer patients [20, 22, 24, 28-29]. Although unable to provide definitive causation, these studies nonetheless present compelling evidence for the potential for wait times to adversely affect therapeutic outcomes.

The median time interval of 65.5 d from abnormal image to treatment initiation in this study falls well within the range of values of previous reports, which vary from a high of 73 to 112 d [8, 14, 30-31] in one set of studies to a low of 20 to 35 d in another other [20-21]. The Swedish Lung Cancer Study Group has recommended initiation of treatment within 42 d of abnormal imaging [32], whereas an independent report commissioned by the RAND Corporation has advocated treatment initiation within 42 d of diagnosis [33]. In Canada, guidelines from Cancer Care Ontario has recommended a maximum of 21 d from initial presentation to a general practitioner to referral to a specialist [34], with initiation of treatment within 28 d of a cancer diagnosis [35].

### Limitations and strengths

In interpreting the results of this study, several limitations must be noted. These include the study’s small sample size and higher than anticipated exclusion rate, contributing to a possible patient selection bias. In addition, an important time interval not assessed here is the time elapsed from initial symptoms to the first presentation to a medical practitioner due to a significant variability in physician records and subjectivity of presenting complaints. However, this interval constitutes a significant component of the care pathway, with delays reported from 14 d in Finland, 21 d in Ontario, and 43 d in the United States [23, 30, 36].

An important endpoint not addressed in this study is how overall survival correlates to wait times experienced. This parameter requires a larger study population to have sufficient power to investigate this important relationship. This will form the basis for a future province-wide study. Additionally, the delay from imaging to treatment on survival between NSCLC and SCLC subgroups could also be addressed in a future study with a larger population.

### Implications for practice

Any delay between the onset of symptoms, diagnosis, and oncologic treatment may permit significant disease progression and thereby negating a subset of patients the opportunity for curative intent [8-9]. For instance, tumour size has been reported to increase by up to 373% between diagnostic and planning CT scans, constituting a median interval of 54 d [8], during which a fraction of patients were deemed ineligible for curative treatment. Further support comes from the finding that patients who waited greater than four months for diagnosis following an abnormal image had a worse outcome than those diagnosed sooner [9]. Together, these studies provide further support for the notion that delays in care confer a worse prognosis.

In agreement with previous studies, an interval of 43 d (median) from the first abnormal image to referral to BCCA-CSI suggests that most delays occur during patient diagnosis and staging [18-19]. Accordingly, previous efforts to reduce delays have targeted these areas of patient management. Through restructuring of referral patterns, a thoracic surgical centre reduced the time interval from the decision to treat to initiation of treatment from 38 to 8 d [37]. Similarly, a randomized controlled trial obtained a ~50% reduction in time from the first presentation to treatment by devising a multidisciplinary-based treatment plan within 3 d of initial imaging tests and biopsy [38]. Accordingly, successful surgical resection rates more than doubled as compared to historic controls [38-39].

Perhaps the most successful interventions to date have involved streamlining the referral process. A redesign of this process featuring the addition of a nurse navigator at the Surrey Memorial Hospital termed the Rapid Autopilot Program (RAP) has reduced surgical wait times for lung cancer patients from 190 to 45 d [40]. Similarly, the Time To Treat Program implemented at the Toronto East General Hospital in Toronto, Ontario has enjoyed tremendous success. This latter program incorporates a clerical navigator in a streamlined referral system, reducing the time interval from suspicion of lung cancer to diagnosis from 128 to 20 d [ 21]. By comparison, our study revealed a median wait time from first abnormal image to cancer centre referral (assumed date of diagnosis) of 43 d. Although, direct comparisons between studies are confounded due to differences in defining time intervals, at present patients in the BC Southern Interior continue to experience delays that may determine whether a case is surgically resectable, amenable to chemotherapy and/or radiation therapy with curative intent, or treatable by palliative measures only. For instance, 25% of patients treated with curative intent at BCCA-CSI waited greater than 133 d to initiate cancer treatment. Considering small cell lung cancer exhibits a doubling-time as short as 30 days, there is potential for significant disease progression during this 4.5-month wait period.

## Conclusions

We report significant time delays experienced by lung cancer patients during their cancer care path from diagnostic imaging to initiation of oncologic treatment (median 65.5 d). Moreover, 25% of patients waited longer than 104.3 d. While waiting for care, there is potential for significant disease progression, and tragically, a subset of patients may no longer be eligible for curative intent treatment. Moreover, we provide evidence that rural patients, on average, access care more promptly than their urban counterparts. This study provides data to support the need for operational change and suggests basic interventions to help reduce bottlenecks relevant to the Canadian cancer care system. 
